# Modeling of COVID-19 vaccination rate using odd Lomax inverted Nadarajah-Haghighi distribution

**DOI:** 10.1371/journal.pone.0276181

**Published:** 2022-10-21

**Authors:** Hisham M. Almongy, Ehab M. Almetwally, Hanan Haj Ahmad, Abdullah H. Al-nefaie

**Affiliations:** 1 Applied Statistics & Insurance Department, Faculty of Commerce Mansoura University, Mansoura, Egypt; 2 Department of Statistics, Faculty of Business Administration, Delta University of Science and Technology, Gamasa, Egypt; 3 The Scientific Association for Studies and Applied Research, Al Manzalah, Egypt; 4 Department of Basic Science, Preparatory Year Deanship, King Faisal University, Hofuf, Al-Ahsa, Saudi Arabia; 5 Quantitative Methods Department, College of Business, King Faisal University, Al Ahsa, Saudi Arabia; Universita degli Studi di Milano, ITALY

## Abstract

Since the spread of COVID-19 pandemic in early 2020, modeling the related factors became mandatory, requiring new families of statistical distributions to be formulated. In the present paper we are interested in modeling the vaccination rate in some African countries. The recorded data in these countries show less vaccination rate, which will affect the spread of new active cases and will increase the mortality rate. A new extension of the inverted Nadarajah-Haghighi distribution is considered, which has four parameters and is obtained by combining the inverted Nadarajah-Haghighi distribution and the odd Lomax-*G* family. The proposed distribution is called the odd Lomax inverted Nadarajah-Haghighi (OLINH) distribution. This distribution owns many virtuous characteristics and attractive statistical properties, such as, the simple linear representation of density function, the flexibility of the hazard rate curve and the odd ratio of failure, in addition to other properties related to quantile, the *r*^*th*^-moment, moment generating function, Rényi entropy, and the function of ordered statistics. In this paper we address the problem of parameter estimation from frequentest and Bayesian approach, accordingly a comparison between the performance of the two estimation methods is implemented using simulation analysis and some numerical techniques. Finally different goodness of fit measures are used for modeling the COVID-19 vaccination rate, which proves the suitability of the OLINH distribution over other competitive distributions.

## 1 Introduction

The amount of data obtained for analysis has been growing increasingly, requiring new statistical distributions that enables us to depict every phenomenon under study. Modeling real-life observations using probability distributions is one of the most essential responsibilities that statisticians must handle. Many scientific fields require statistical models to describe the trend and to predict the future behaviour of their data, for example, medical, engineering, finance, and others. Therefore many lifetime models have been employed in literature to describe various forms of survival data, so the newly created families of distributions are strongly depending on the quality of statistical analysis processes,the flexibility and the characteristics of the new models, therefore, significant efforts are focusing on constructing new statistical models. Still there is a persistent need to create new models or formulate new extensions for achieving better fit of the real lifetime data.

Tahir et al. [1] proposed the inverted Nadarajah-Haghighi (INH)) distribution,which is a new inverted model with decreasing and uni-modal (right-skewed) density, with decreasing and upside-down bathtub hazard rate shapes (UBT). They addressed several statistical features of the INH distribution and used various frequentest approaches to estimate the model’s parameters. They have demonstrated the suitability of INH distribution by testing real-life data sets. They also obtained that the INH model was better fit with comparison to other well-known lifetime models such as, the inverted exponential, the inverted gamma, the inverted Weibull and the inverted Lindley among others.

Several researchers have addressed the applications of inverted distributions, one can refer to Folks and Chhikara [2], Rosaiah and Kantam [3], De Gusmao et al. [4], Joshi and Kumar [5], Almetwally [6], Ibrahim and Almetwally [7], Ramos et al. [8], Almetwally [9], Hassan et al. [10], and Basheer et. al [11] among others. Some generalizations of the INH distribution were introduced in literature for example, the Marshall-Olkin INH distribution was studied by Raffiq et al. [12], Toumaj et al. [13] proposed the transmuted INH distribution. Elshahhat and Rastogi [14] discussed parameter estimation of lifetime for the INH distribution with Type-II progressively censored samples. Still there is space for new generalizations and extensions for the INH distribution, consequently, the new extension is superior to the original INH distribution and other competitive models specially for modeling COVID-19 vaccination rate.

Let x be a random variable with the parameters *δ*, *θ* > 0 that follows the inverse Nadarajah-Haghighi distribution (INH). The CDF and *pdf* are as follows:

$$\begin{array}{c} G\left(x;\Theta \right)=e^{1-{\left(1+\frac{\delta }{x}\right)}^{\theta }};\;\;\;\;\;\;\;x>0,\delta ,\theta >0 \end{array}$$ (1)

and,

$$\begin{array}{c} g\left(x;\Theta \right)=\frac{\delta \theta }{x^{2}}{\left(1+\frac{\delta }{x}\right)}^{\theta -1}e^{1-{\left(1+\frac{\delta }{x}\right)}^{\theta }};\;\;\;\;\;x,\delta ,\theta >0, \end{array}$$ (2)

where Θ = (*δ*, *θ*) is a parameter vector of INH distribution.

In this work we are introducing a new extension of INH distribution with four parameters, namely the odd Lomax INH (OLINH) based on the odd Lomax-*G* (OL-G) family introduced by Cordeiro et al. [15]. Adding more parameters to the original distribution improves that distribution and make it more flexible and reliable to model some real life data.

Let $g(x;\Theta )=\frac{dG(x;\Theta )}{dx}$ be the *pdf* of a baseline model with vector parameter Θ, then the CDF of the OL-G family is given by:

$$\begin{array}{c} F\left(x;\Omega \right)=1-\beta^{\alpha }{\left[\beta +\frac{G(x;\Theta )}{1-G(x;\Theta )}\right]}^{-\alpha },x>0,\alpha ,\beta >0, \end{array}$$ (3)

where Ω = (*α*, *β*, Θ) is a vector of parameters of OL-G family. The *pdf* of (3) is defined by

$$\begin{array}{c} f\left(x;\Omega \right)=\frac{\alpha \beta^{\alpha }g\left(x;\Theta \right)}{{\left(1-G\left(x;\Theta \right)\right)}^{2}}{\left[\beta +\frac{G(x;\Theta )}{1-G(x;\Theta )}\right]}^{-\alpha -1} \end{array}$$ (4)

where *α*, *β* > 0 are shape parameters. The random variable with *pdf* (4) is denoted by *X* ∼OL-G(Ω). A new extended four-parameter Weibull, Lomax, log-logistic, and log-Lindley distributions, called the OL-Weibull, OL-Lomax, OL-log-logistic, and OL-log-Lindley distributions respectively, were introduced by Cordeiro et al. [15]. Odd Lomax-exponential distribution was introduced by Ogunsanya et al. [16]. Yakura et al. [17] introduced the Lomax-Kumaraswamy distribution. Marzouk et al [18] obtained a generalized odd Lomax family of distributions with applications. The extended odd Lomax family of distribution was described by Abubakari et al. [19].

The main idea of this work is to study the statistical properties of the new extension model and investigate the point and interval estimation for its unknown four-parameters. Two estimation methods are considered: the maximum likelihood, and the Bayesian estimation methods. To verify the efficiency of the proposed estimation methods and to study how these estimators perform for various sample sizes and parameter values, statistical analysis is carried out using simulation study via R-coding. A real data example emphasizes the suitability of OLINH model over INH and other competitive models with two and three parameters. The rest of this article is organized as follows: The OLINH distribution is defined in Section 2. In Section 3, some statistical properties for the OLINH distribution are obtained. Section 4 studies two methods of estimation. To judge the efficiency of these estimation methods, a simulation study is performed in Section 5. The Application of COVID-19 vaccinate rate data from 46 different African countries is considered in Section 6 for illustrative purpose. Finally, in Section 7, conclusions are provided.

## 2 OLINH distribution

Consider the OL-G family with the INH distribution as a baseline function, then a four-parameters OLINH distribution is generated. By substituting the INH model’s CDF and *pdf* files (1) and (2) in the OL-G family (3) and (4), the OLINH distribution CDF and *pdf* are obtained as:

$$\begin{array}{c} F\left(x;\Omega \right)=1-\beta^{\alpha }{\left[\beta +\frac{e^{1-{\left(1+\frac{\delta }{x}\right)}^{\theta }}}{1-e^{1-{\left(1+\frac{\delta }{x}\right)}^{\theta }}}\right]}^{-\alpha }, \end{array}$$ (5)

and

$$\begin{array}{c} f\left(x;\Omega \right)=\alpha \theta \beta^{\alpha }\frac{\frac{\delta }{x^{2}}{\left(1+\frac{\delta }{x}\right)}^{\theta -1}e^{1-{\left(1+\frac{\delta }{x}\right)}^{\theta }}}{{\left(1-e^{1-{\left(1+\frac{\delta }{x}\right)}^{\theta }}\right)}^{2}}{\left[\beta +\frac{e^{1-{\left(1+\frac{\delta }{x}\right)}^{\theta }}}{1-e^{1-{\left(1+\frac{\delta }{x}\right)}^{\theta }}}\right]}^{-\alpha -1}, \end{array}$$ (6)

respectively, where *x* > 0, *α*, *β*, *δ*, *θ* > 0. A random variable with *pdf* (6) is denoted by *X* ∼OLINH(*α*, *β*, *δ*, *θ*). The hazard rate function (*hrf*) of the OLINH distribution is given by

$$\begin{array}{c} h\left(x;\Omega \right)=\alpha \delta \theta \frac{1}{x^{2}}{\left(1+\frac{\delta }{x}\right)}^{\theta -1}\frac{{\left[1-e^{1-{\left(1+\frac{\delta }{x}\right)}^{\theta }}\right]}^{-1}}{\beta \left[e^{-1+{\left(1+\frac{\delta }{x}\right)}^{\theta }}-1\right]+1}. \end{array}$$

The odds ratio of failure (ORF) of the OLINH distribution is otained by

$$\begin{array}{c} ORF\left(x;\Omega \right)=\beta^{-\alpha }{\left[\beta +\frac{e^{1-{\left(1+\frac{\delta }{x}\right)}^{\theta }}}{1-e^{1-{\left(1+\frac{\delta }{x}\right)}^{\theta }}}\right]}^{\alpha }-1. \end{array}$$

**Fig 1:**
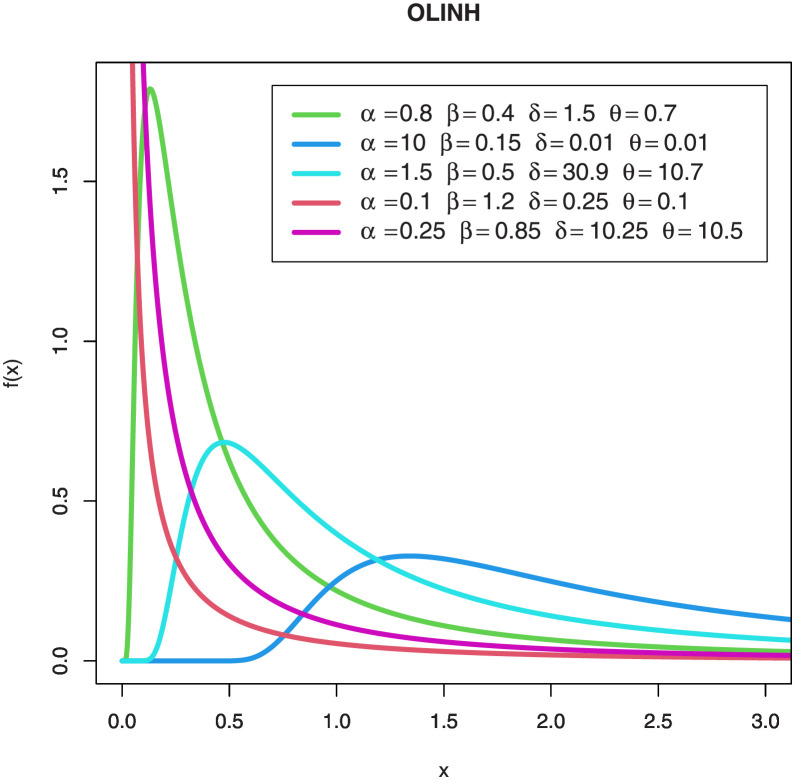
Plots of the OLINH densities for some parameter values.

**Fig 2:**
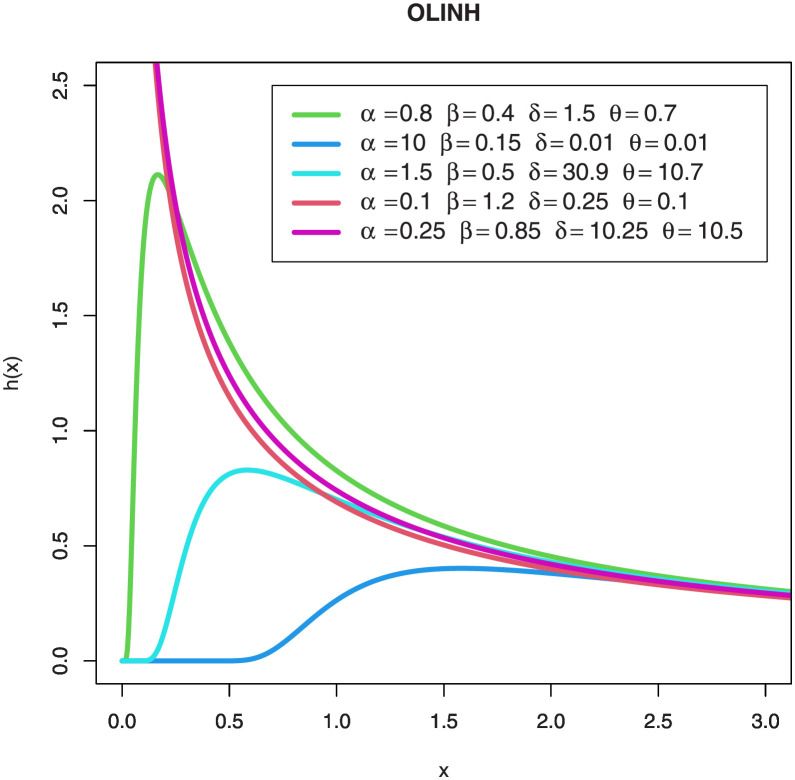
Plots of the *hrf* of the OLINH distribution for some parameter values.

Figs 1 and 2 are separate shapes of the OLINH distribution’s *pdf* and the *hrf* for different parameters values respectively. The density shape of the OLINH distribution can be right-skewed and Rev-J shaped. The *hrf* of the OLINH distribution has some interesting shapes, such as, decreasing and upside down bathtub. Different shapes of *hrf* create an appealing features for modeling many lifetime data such as biomedical and biological studies, reliability analysis, physical engineering, and survival analysis.

## 3 Statistical characteristics of OLINH distribution

In this section, we observe some statistical characteristics of the OLINH distribution, such as, the linear representation of its *pdf*, quantile, the moments, the moment generating function (MGF), Rényi entropy and ordered statistic function.

### 3.1 Linear representation

According to Cordeiro et al. [15] the linear representation for the density of the OL-G family is given by

$$\begin{array}{c} f\left(x,\Omega \right)=\sum_{k,j=0}^{\infty }\Delta_{k,j}\left(k+j+1\right)g\left(x,\Theta \right)G{\left(x,\Theta \right)}^{k+j}, \end{array}$$ (7)

where Δk,j=(-1)jα(k+j+1)βk+1(-α-1k)(-k-2j). The linear representation for the cumulative density of the OL-G family is as follows

$$\begin{array}{c} F\left(x,\Omega \right)=\sum_{k,j=0}^{\infty }\Delta_{k,j}G{\left(x,\Theta \right)}^{k+j+1}. \end{array}$$ (8)

Using Eq (7), the Linear representation for the *pdf* of the OLINH density can be written as

$$\begin{array}{c} f\left(x,\Omega \right)=\sum_{k,j=0}^{\infty }\Delta_{k,j}\left(k+j+1\right)\frac{\delta \theta }{x^{2}}{\left(1+\frac{\delta }{x}\right)}^{\theta -1}e^{\left(k+j+1\right){\left(1-(1+\frac{\delta }{x}\right)}^{\theta }}. \end{array}$$ (9)

Eq (9) denotes the exponentiated INH density with power (*k* + *j* + 1). Using Eq (8), we obtain the linear representation of CDF for the OLINH distribution

$$\begin{array}{c} F\left(x,\Omega \right)=\sum_{k,j=0}^{\infty }\Delta_{k,j}\left(k+j+1\right)e^{\left(k+j+1\right){\left(1-(1+\frac{\delta }{x}\right)}^{\theta }}. \end{array}$$ (10)

Linear representation for *pdf* and CDF of the OLINH are valuable when finding moments, moment generating function, Rényi entropy, and ordered statistics density.

### 3.2 Quantile for the OLINH distribution

The quantile of a certain distribution is an important measure of location, it is usually used to create a random sample in simulation analysis. To do so, let *x* = *Q*(*x*) = *F*(*x*, Ω)^−1^, hence for the OLINH distribution the quantile can be obtained by inverting Eq (5) to get:

$$\begin{array}{c} x_{q}=\delta \frac{1}{{\left(1+ln\left[1+\frac{1}{\beta \left({\left(1-q\right)}^{\frac{-1}{\alpha }-1}\right)}\right]\right)}^{\frac{1}{\theta }-1}};\;0<q<1 \end{array}$$ (11)

**Fig 3:**
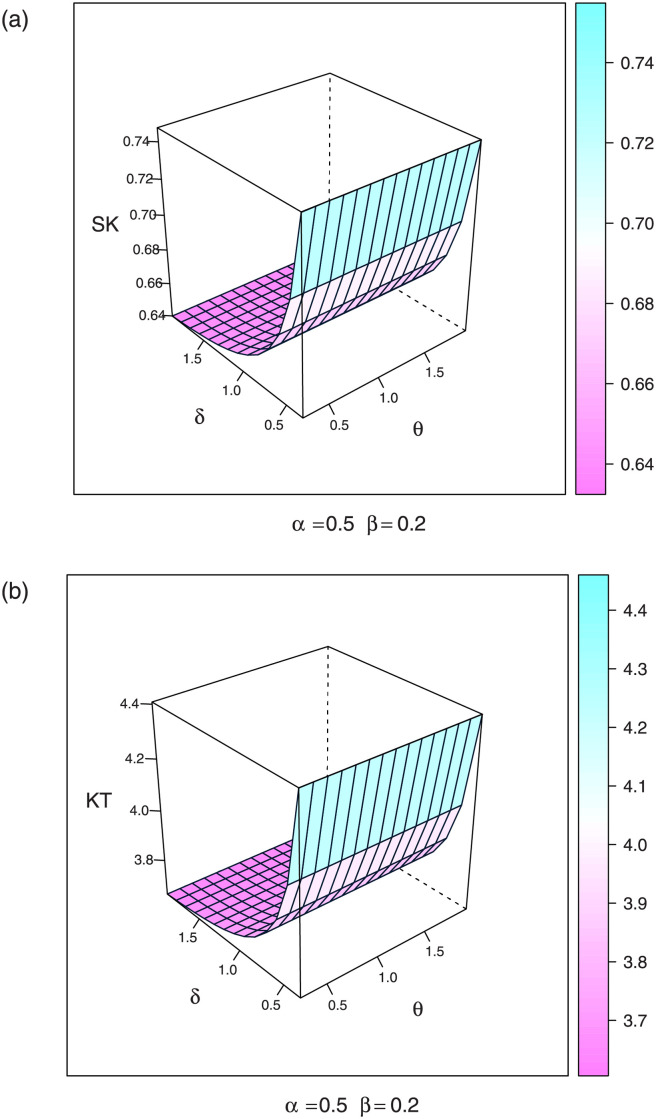
Plots of skewness and kurtosis values the OLINH for some parameter values.

In particular, the three quartiles, say Q1, Q2, and Q3 can be observed by selecting some fixed values of *q* = 0.25, 0.5, and 0.75, respectively, in Eq (11). By this equation, we can obtain skewness and kurtosis measures, see Fig 3.

### 3.3 Moments for the OLINH distribution

Let *x* be a random variable following the OLINH distribution, then the *r*^*th*^ moment of *x* follows from Eq (9), and using power series with some algebraic manipulations to have the following

$$\begin{array}{cc} \overset{´}{\mu_{r}} & =E\left(x^{r}\right)\\ & =\sum_{i,k,j=0}^{\infty }\Delta_{k,j}a_{i}\left(r\right){\left(k+j+1\right)}^{-i/\theta }e^{(k+j+1)}\Gamma \left(\frac{i}{\theta }+1,1\right) \end{array}$$

where $a_{i}\left(r\right)=\delta^{r}\frac{{\left(-1\right)}^{r+i+1}}{i!}\prod_{s=0}^{\infty }\left(r+s\right).$ The ordinary moments are useful in evaluating skewness and kurtosis values see Fig 3. The *r*^*th*^ incomplete moment of OLINH is expressed as

$$\begin{array}{cc} m_{r}\left(y\right) & =\int_{0}^{y}x^{r}f\left(x\right)dx\\ & =\sum_{i,j,k0}^{\infty }\Delta_{k,j}a_{i}\left(r\right){\left(k+j+1\right)}^{-i/\theta }e^{k+j+1}\gamma \left(\frac{i}{\theta }+1,y\right) \end{array}$$

where $\gamma \left(b,y\right)=\int_{0}^{y}z^{b-1}e^{-z}dz$ is the lower incomplete gamma function. The incomplete moment is useful in finding Benferroni and Lorenz curves, mean residual life, mean waiting time and other measures.

The moment generating function of OLINH distribution is given by

$$\begin{array}{cc} \overset{´}{M_{x\left(t\right)}} & =E\left(e^{xt}\right)\\ & =\sum_{i,k,j=0}^{\infty }\Delta_{k,j}\sum_{m=0}^{\infty }\frac{t^{m}}{m!}{\left(k+j+1\right)}^{\frac{-i}{\theta }}e^{k+j+1}\gamma \left(\frac{i}{\theta }+1,1\right). \end{array}$$ (12)

### 3.4 Rényi entropy

Rényi entropy is known as an extension of Shannon entropy, Rényi entropy of order *ζ* is defined as

$$\begin{array}{c} I_{\zeta }\left(x\right)=\frac{1}{1-\zeta }\text{log}\;\left(\int_{-\infty }^{\infty }f{\left(x\right)}^{\zeta }dx\right),\;\;\;\;\zeta >0,\zeta \neq 1 \end{array}$$

Using the OLINH density from Eq (6), and apply the power series with integration techniques and some algebraic simplification Rényi entropy can be written as

Iζ(x)=11-ζlog[δθ2ζ-1∑l,n,s=0∞Rl,n(-1)s+ne(ζ+n+l)(ζ+n+l)-s+ζ(θ-1)θ(2(ζ-1)s)Γ(s+ζ(θ-1)θ+1)] (13)

where Rl,n=β-l(αβ)ζ(-2ζ-ln)(-ζ(α+1)l).

**Fig 4:**
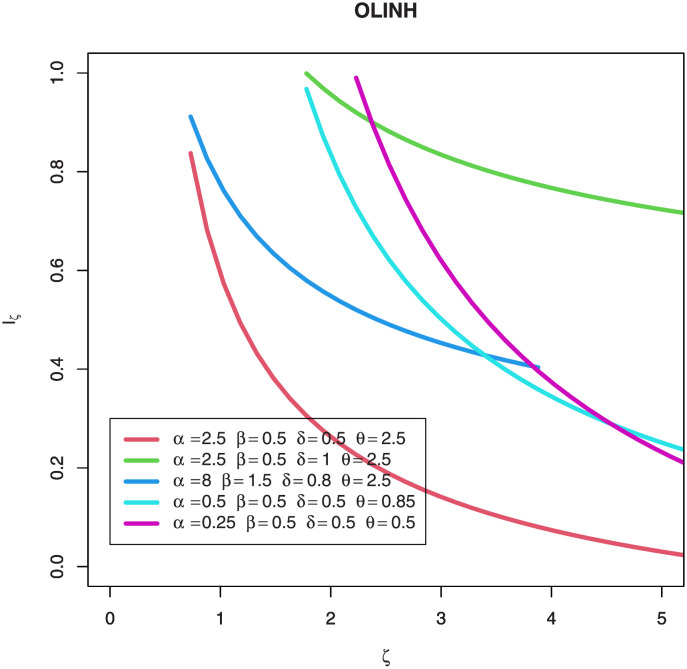
Plots of the OLINH Rényi entropy for some parameter values.

Fig 4 shows the Rényi Entropy for some parameter values of OLINH model with different values of *ζ*. Rényi Entropy has many applications for more information see [20–23]. By this Figure, we note that the Rényi Entropy decreases when the *ζ* values increases.

### 3.5 Order statistics

Let *x*_1_, …, *x*_*n*_ be a sample of size *n* drawn randomly from a continuous pdf *f*(*x*). Suppose *x*_1:*n*_ < *x*_2:*n*_ < … < *x*_*n*:*n*_ are the related order statistics. If the random sample follow OLINH distribution, then from Eqs (8) and (9) the *pdf* of the *k*^*th*^ order statistics *x*_*k*:*n*_ is given by

$$\begin{array}{c} f_{k:n}\left(x\right)=\sum_{t,u=0}^{\infty }b_{t,u}h_{t+u+1}\left(x\right) \end{array}$$

where *h*_*t*+*u*+1_(*x*) is the exponentiated INH density with power *t* + *u* + 1 and

bt,u=∑j=0n-k∑m=0j+k-1(-1)m+uαβ-t-1B(k,n-k+1)(n-kj)(-t-2u)(j+k-1m)(-(m+1)α-1t)

From the above equation we can say that the OLINH order statistics *pdf* is a represented as a linear combination of the exponentiated INH densities, hence many statistical properties of the ordered statistics can be derived easily from the characteristic of *h*_*t*+*u*+1_(*x*).

## 4 Estimation methods

The estimation problem of the OLINH distribution parameters is studied in this section using: The maximum likelihood estimator (MLE), and the Bayesian estimation based on the squared error loss function.

### 4.1 Maximum likelihood estimation

Let *X*_1_, …, *X*_*n*_ be a random sample from OLINH distribution with parameters *α*, *β*, *δ* and *θ*. Then the log-likelihood for the OLINH is provided by

$$l\left(\Omega \right)=n\left[\;\text{ln}\left(\alpha \right)+\;\text{ln}\left(\theta \right)+\alpha \;\text{ln}\left(\beta \right)+\;\text{ln}\left(\delta \right)\right]-2\sum_{i=1}^{n}ln\left(x_{i}\right)+\left(\theta -1\right)\sum_{i=1}^{n}\;\text{ln}\left(1+\frac{\delta }{x_{i}}\right)+\sum_{i=1}^{n}\left[1-{\left(1+\frac{\delta }{x_{i}}\right)}^{\theta }\right]-2\sum_{i=1}^{n}\;\text{ln}\left[1-e^{1-{\left(\frac{1+\delta }{x}\right)}^{\theta }}\right]-\left(\alpha +1\right)\sum_{i=1}^{n}\;\text{ln}\left[\beta +\frac{e^{1-{\left(1+\frac{\delta }{x_{i}}\right)}^{\theta }}}{1-e^{1-{\left(1+\frac{\delta }{x_{i}}\right)}^{\theta }}}\right]$$ (14)

To maximize the log-likelihood equation, we need to take the partial derivatives of *l*(Ω) with respect to the model parameters *α*, *β*, *δ* and *θ* and equate them to zero, hence we obtain the following system of nonlinear equations:

$$\frac{\partial l\left(\Omega \right)}{\partial \alpha }=\frac{n}{\alpha }+n\;\text{ln}\left(\beta \right)-\sum_{i=1}^{n}\;\text{ln}\left[\beta +\frac{e^{1-{\left(1+\frac{\delta }{x_{i}}\right)}^{\theta }}}{1-e^{1-{\left(1+\frac{\delta }{x_{i}}\right)}^{\theta }}}\right]=0$$ (15)

$$\frac{\partial l(\Omega )}{\partial \beta }=\frac{n\alpha }{\beta }-(\alpha +1)\sum_{i=1}^{n}{\left[\beta +\frac{e^{1-{\left(1+\frac{\delta }{x_{i}}\right)}^{\theta }}}{1-e^{1-{\left(1+\frac{\delta }{x_{i}}\right)}^{\theta }}}\right]}^{-1}=0$$ (16)

$$\frac{\partial l(\Omega )}{\partial \delta }=\frac{n}{\delta }+(\theta -1)\sum_{i=1}^{n}\frac{\frac{1}{x_{i}}}{1+\frac{\delta }{x_{i}}}-\theta \sum_{i=1}^{n}A_{i}(\delta ,\theta )-2\theta \sum_{i=1}^{n}\frac{A_{i}(\delta ,\theta )e^{1-{\left(1+\frac{\delta }{x_{i}}\right)}^{\theta }}}{1-e^{1-{\left(1+\frac{\delta }{x_{i}}\right)}^{\theta }}}+(\alpha +1)\sum_{i=1}^{n}\frac{A_{i}(\delta ,\theta )e^{1-{\left(1+\frac{\delta }{x_{i}}\right)}^{\theta }}}{\left[\beta +(1-\beta )e^{1-{\left(1+\frac{\delta }{x_{i}}\right)}^{\theta }}\right]\left[1-e^{1-{\left(1+\frac{\delta }{x_{i}}\right)}^{\theta }}\right]}=0$$ (17)

and

$$\frac{\partial l(\Omega )}{\partial \delta }=\frac{n}{\theta }+\sum_{i=1}^{n}\;\text{ln}\left(1+\frac{\delta }{x_{i}}\right)-\sum_{i=1}^{n}B_{i}(\delta ,\theta )-2\sum_{i=1}^{n}\frac{B_{i}(\delta ,\theta )e^{1-{\left(\frac{1+\delta }{x}\right)}^{\theta }}}{1-e^{1-{\left(\frac{1+\delta }{x}\right)}^{\theta }}}+(\alpha +1)\sum_{i=1}^{n}\frac{B_{i}(\delta ,\theta )e^{1-{\left(\frac{1+\delta }{x}\right)}^{\theta }}}{\left[\beta +(1-\beta )e^{1-{\left(\frac{1+\delta }{x}\right)}^{\theta }}\right]\left[1-e^{1-{\left(\frac{1+\delta }{x}\right)}^{\theta }}\right]}=0$$ (18)

where $A_{i}\left(\delta ,\theta \right)=\frac{1}{x_{i}}{\left(1+\frac{\delta }{x_{i}}\right)}^{\theta -1}$ and $B_{i}\left(\delta ,\theta \right)={\left(1+\frac{\delta }{x_{i}}\right)}^{\theta }\text{ln}(1+\frac{\delta }{x_{i}}).$ It is possible to obtain the MLE of *α* ($\hat{\alpha }$) explicitly from Eq (15), hence

$$\begin{array}{c} \hat{\alpha }=\frac{n}{-n\;\text{ln}\;\hat{\beta }+\sum_{i=1}^{n}\text{ln}\left[\hat{\beta }+\frac{e^{1-{\left(1,+,\frac{\hat{\delta }}{x}\right)}^{\hat{\theta }}}}{1-e^{1-{\left(1,+,\frac{\hat{\delta }}{x}\right)}^{\hat{\theta }}}}\right]} \end{array}$$

where $\hat{\beta },\hat{\delta }$ and $\hat{\theta }$, are the MLEs of *β*, *δ* and *θ* respectively, and they are obtained numerically by solving the above system using some techniques such as the Newton-Raphson method, R packages are used for that purpose.

### 4.2 Bayesian estimation

The Bayesian approach deals with the parameters as random variables with certain prior distribution. The ability to incorporate prior knowledge into research makes the Bayesian method very useful in the survival analysis. One of the main problems associated with survival analysis is the limitation of data availability. For the parameters *α*, *β*, *δ* and *θ* we suggest gamma distribution as prior functions, therefore the parameters *α*, *β*, *δ* and *θ* have gamma distributions *Gamma*(*μ*_1_, *ν*_1_), *Gamma*(*μ*_2_, *ν*_2_), *Gamma*(*μ*_3_, *ν*_3_), and *Gamma*(*μ*_4_, *ν*_4_) respectively. Hence the independent joint prior density function can be written as follows:

$$\begin{array}{c} \Pi \left(\Omega \right)\propto \alpha^{\mu_{1}-1}\beta^{\mu_{2}-1}\delta^{\mu_{3}-1}\theta^{\mu_{4}-1}e^{-(\nu_{1}\alpha +\nu_{2}\beta +\nu_{3}\delta +\nu_{4}\theta )} \end{array}$$ (19)

The joint posterior density function of Ω is calculated using the likelihood function and joint prior function, and is given by

$$\prod (\Omega |x)\propto \alpha^{n+\mu_{1}-1}\theta^{n+\mu_{4}-1}\beta^{n\alpha +\mu_{2}-1}\delta^{n+\mu_{3}-1}\prod_{i=1}^{n}\frac{{\left(1+\frac{\delta }{x_{i}}\right)}^{\theta -1}}{{\left(1-e^{1-{\left(\frac{1+\delta }{x_{i}}\right)}^{\theta }}\right)}^{2}}{\left[\beta +\frac{e^{1-{\left(1+\frac{\delta }{x_{i}}\right)}^{\theta }}}{1-e^{1-{\left(1+\frac{\delta }{x_{i}}\right)}^{\theta }}}\right]}^{-\alpha -1}e^{-(\nu_{1}\alpha +\nu_{2}\beta +\nu_{3}\delta +\nu_{4}\theta )}e^{-\sum_{i=1}^{n}{\left(1+\frac{\delta }{x_{i}}\right)}^{\theta }},$$ (20)

Based on the squared error loss function, the Bayes estimators of $\tilde{\Omega }$ is:

$$\begin{array}{cc} S\left(\tilde{\Omega }\right)= & E{\left(\tilde{\Omega }-\Omega \right)}^{2}\\ & \int_{0}^{\infty }\int_{0}^{\infty }\int_{0}^{\infty }\int_{0}^{\infty }{\left(\tilde{\Omega }-\Omega \right)}^{2}\Pi (\Omega |x)d\alpha d\beta d\delta d\theta \end{array}$$ (21)

It’s worth noting that the integrals offered by Eq (21) can’t be obtained manually. As a result, we use a numerical method called the Markov Chain Monte Carlo (MCMC) method to approximate the integrals value. The MCMC method’s most popular applications are the Metropolis-Hasting (MH) algorithm and the Gibbs sampling. The MH algorithm, like acceptance-rejection sampling, assumes that for each iteration of the process, a selected value from a proposal distribution can be generated. We apply the MH inside Gibbs sampling to create random samples of conditional posterior densities from the OLINH distribution family. The posterior conditional distributions are as follows:

$$\begin{array}{c} \Pi (\alpha |\beta ,\delta ,\theta ,x)\propto \alpha^{n+\mu_{1}-1}e^{-\alpha \left[\nu_{1}+\sum_{i=1}^{n}\;\text{ln}\left(\beta +\frac{e^{1-{\left(1+\frac{\delta }{x_{i}}\right)}^{\theta }}}{1-e^{1-{\left(1+\frac{\delta }{x_{i}}\right)}^{\theta }}}\right)\right]},\\ \alpha ∼Gamma\left[n+\mu_{1}\text{,}\nu_{1}+\overset{n}{\underset{i=1}{\sum }}\;\text{ln}\left(\beta +\frac{e^{1-{\left(1+\frac{\delta }{x_{i}}\right)}^{\theta }}}{1-e^{1-{\left(1+\frac{\delta }{x_{i}}\right)}^{\theta }}}\right)\right] \end{array}$$ (22)

$$\begin{array}{c} \Pi (\beta |\alpha ,\delta ,\theta ,x)\propto \beta^{n\alpha +\mu_{2}-1}\overset{n}{\underset{i=1}{\prod }}{\left[\beta +\frac{e^{1-{\left(1+\frac{\delta }{x_{i}}\right)}^{\theta }}}{1-e^{1-{\left(1+\frac{\delta }{x_{i}}\right)}^{\theta }}}\right]}^{-\alpha -1}e^{-\nu_{2}\beta } \end{array},$$ (23)

$$\begin{array}{c} \Pi (\delta |\alpha ,\beta ,\theta ,x)\propto \delta^{n+\mu_{3}-1}e^{-\nu_{3}\delta }e^{-\sum_{i=1}^{n}{\left(1+\frac{\delta }{x_{i}}\right)}^{\theta }}\overset{n}{\underset{i=1}{\prod }}\frac{{\left(1+\frac{\delta }{x_{i}}\right)}^{\theta -1}}{{\left(1-e^{1-{\left(\frac{1+\delta }{x_{i}}\right)}^{\theta }}\right)}^{2}}{\left[\beta +\frac{e^{1-{\left(1+\frac{\delta }{x_{i}}\right)}^{\theta }}}{1-e^{1-{\left(1+\frac{\delta }{x_{i}}\right)}^{\theta }}}\right]}^{-\alpha -1} \end{array},$$ (24)

and

$$\begin{array}{cc} \Pi & (\theta |\alpha ,\beta ,\delta ,x)\propto \theta^{n+\mu_{4}-1}e^{-\nu_{4}\theta }e^{-\sum_{i=1}^{n}{\left(1+\frac{\delta }{x_{i}}\right)}^{\theta }}\overset{n}{\underset{i=1}{\prod }}\frac{{\left(1+\frac{\delta }{x_{i}}\right)}^{\theta -1}}{{\left(1-e^{1-{\left(\frac{1+\delta }{x_{i}}\right)}^{\theta }}\right)}^{2}}{\left[\beta +\frac{e^{1-{\left(1\text{+}\frac{\delta }{x_{i}}\right)}^{\theta }}}{1-e^{1-{\left(1+\frac{\delta }{x_{i}}\right)}^{\theta }}}\right]}^{-\alpha -1}. \end{array}$$ (25)

## 5 Simulation

In this section, the Monte-Carlo simulation process is utilized to compare the conventional estimation methods: MLE and Bayesian estimation method under square error loss function. Simulation analysis is based on MCMC method for estimating the OLINH lifespan distribution’s parameters using R software with 5000 iterations, hence random samples are generated from the OLINH distribution samples, where *x* represents the OLINH lifetime for various parameter actual values and sample sizes n: (30, 80, and 150). Different real values of the parameters of the OLINH distribution are obtained.

Asymptotic confidence intervals for MLE and the Bayesian credible intervals are obtained, the highest posterior density interval (HPDI) was used for finding the credible intervals. The best estimator method is defined by minimizing estimator’s relative bias (RB), the mean squared error (MSE), and the length of confidence interval (L.CI).

$RB\left(\Omega \right)=\frac{\frac{1}{N}\sum_{1}^{N}{\hat{\Omega }}_{j}-\Omega }{\Omega }$ , $MSE\left(\Omega \right)=\frac{1}{N}{\left(\sum_{1}^{N}{\hat{\Omega }}_{j}-\Omega \right)}^{2}$ and *L*.*CI*(Ω) = *Upper*(Ω)−*Lower*(Ω)

Tables 1–3 summarize the simulation results of the methods discussed in this paper for point and interval estimation. The RB, MSE, and L.CI values are used to make the essential comparisons between various point and interval estimating methods. The following conclusions are summarized from these tables:

1. The RB, MSE, and L.CI decrease as *n* increases for actual parameters of the OLINH distribution.
2. Bayesian estimation is the best estimation method.
3. Credible interval of Bayesian estimation by HPDI is the shortest CI of parameters of OLINH distribution.
4. For fixed *α*, *β*, *δ*, and sample size, the RB, MSE, and L.CI increase as *θ* increases.
5. For fixed *α*, *δ*, *θ*, and sample size, the RB, MSE, and L.CI increase as *β* increases, in almost all cases.
6. For fixed *β*, *δ*, *θ*, and sample size, the RB, MSE, and L.CI increase as *α* increases,in almost all cases.

**Table 1:** RB, MSE, and length of CI of OLINH distribution for MLE, and Bayesian when *α* = 1.25, *β* = 1.5, *δ* = 1.5.

*α* = 1.25, *β* = 1.5, *δ* = 1.5									
			MLE				Bayesaian		
*θ*	n		Bias	MSE	L.CI	CP	Bias	MSE	L.CI
0.3	30	*α*	0.1138	0.7438	2.8408	95.80%	-0.1767	0.3758	1.0202
		*β*	0.4749	0.9418	2.4175	93.60%	0.0877	0.3879	1.3903
		*δ*	-0.4943	0.8442	1.5838	92.40%	-0.1720	0.4010	1.1108
		*θ*	0.9478	0.8511	2.7743	96.80%	-0.3469	0.2581	0.6394
	80	*α*	0.0650	0.5690	2.1996	97.20%	-0.1720	0.3206	0.7089
		*β*	0.4509	0.7906	1.6069	95.00%	0.0831	0.2592	0.8553
		*δ*	-0.5736	0.8283	0.7752	93.80%	-0.2205	0.3891	0.7692
		*θ*	0.9215	0.6943	2.0380	96.40%	-0.3743	0.2237	0.4409
	150	*α*	0.0257	0.3329	1.2976	98.20%	-0.1089	0.1984	0.4442
		*β*	0.4102	0.6688	1.0284	97.00%	0.0479	0.1372	0.4435
		*δ*	-0.6099	0.7922	0.4485	95.20%	-0.1753	0.2894	0.4556
		*θ*	0.9106	0.5745	1.3749	97.60%	-0.4495	0.2131	0.2057
1.5	30	*α*	0.2785	1.1166	4.0630	95.40%	-0.0041	0.2997	1.1029
		*β*	0.3516	1.4257	5.1972	96.00%	0.0055	0.3823	1.4560
		*δ*	0.2608	0.7933	2.7079	97.40%	0.0363	0.2954	1.0875
		*θ*	0.0002	0.8658	3.3973	95.80%	0.0012	0.3474	1.2692
	80	*α*	0.0988	0.5452	2.0587	95.40%	-0.0158	0.1883	0.7115
		*β*	0.1555	0.8459	3.1906	95.60%	-0.0096	0.2397	0.9489
		*δ*	0.1222	0.5187	1.9041	96.20%	0.0059	0.1657	0.6515
		*θ*	0.0030	0.6036	2.3685	95.40%	0.0011	0.2173	0.8495
	150	*α*	0.0502	0.3931	1.5141	96.40%	-0.0038	0.1085	0.4265
		*β*	0.1033	0.7078	2.7102	96.80%	-0.0037	0.1308	0.4954
		*δ*	0.1332	0.5084	1.8346	96.40%	0.0051	0.0991	0.3965
		*θ*	-0.0120	0.6043	2.3703	96.20%	-0.0027	0.1163	0.4597
3.5	30	*α*	0.2688	1.2366	4.5872	93.80%	0.0302	0.3026	1.1072
		*β*	0.1029	1.4508	5.6604	93.40%	0.0277	0.3868	1.4855
		*δ*	0.5312	0.9658	2.1415	95.40%	0.1453	0.3412	1.0154
		*θ*	-0.0980	1.0177	3.7598	95.20%	0.0110	0.4370	1.5967
	80	*α*	0.0675	0.6421	2.4879	96.80%	0.0162	0.1907	0.7004
		*β*	-0.1169	0.8402	3.2242	95.40%	-0.0081	0.2436	0.9779
		*δ*	0.4556	0.8206	1.7820	95.40%	0.1338	0.2508	0.5691
		*θ*	-0.0969	0.8877	3.2188	93.60%	0.0136	0.2588	0.9759
	150	*α*	0.0101	0.3482	1.3650	96.00%	-0.0005	0.1046	0.4177
		*β*	-0.2107	0.5770	1.8942	95.40%	0.0061	0.1270	0.4924
		*δ*	0.4091	0.6793	1.1429	95.20%	0.0879	0.1582	0.3397
		*θ*	-0.0939	0.6436	2.1717	94.60%	0.0107	0.1374	0.5276

**Table 2:** RB, MSE, and length of CI of OLINH distribution for MLE, and Bayesian when *α* = 1.25, *δ* = 1.5, *θ* = 1.5.

*α* = 1.25, *δ* = 1.5, *θ* = 1.5									
			MLE				Bayesaian		
*θ*	n		Bias	MSE	L.CI	CP	Bias	MSE	L.CI
0.3	30	*α*	0.3056	1.0132	3.6826	94.80%	0.0122	0.2857	1.0788
		*β*	0.7582	0.5931	2.1493	94.80%	0.3020	0.2152	0.7287
		*δ*	0.1382	0.4022	1.3523	95.80%	0.0344	0.2534	0.9057
		*θ*	-0.0690	0.4626	1.7693	95.60%	-0.0062	0.3474	1.3517
	80	*α*	0.1285	0.5077	1.8900	94.80%	0.0079	0.2055	0.8396
		*β*	0.3738	0.3208	1.1792	94.80%	0.1538	0.1327	0.4787
		*δ*	0.0700	0.2841	1.0357	94.80%	-0.0019	0.1459	0.5681
		*θ*	-0.0467	0.3729	1.4373	94.60%	0.0083	0.2205	0.8806
	150	*α*	0.0597	0.3363	1.2866	94.20%	-0.0072	0.1056	0.4085
		*β*	0.1859	0.2083	0.7877	94.80%	0.0208	0.0724	0.2664
		*δ*	0.0481	0.2070	0.7613	93.60%	0.0062	0.0916	0.3556
		*θ*	-0.0356	0.3005	1.1603	94.40%	-0.0025	0.1130	0.4300
2	30	*α*	0.2573	0.9687	3.5854	93.80%	0.0107	0.2489	0.9260
		*β*	0.2319	1.6410	6.1767	95.60%	0.0046	0.4165	1.5837
		*δ*	0.2761	0.8985	3.1290	98.00%	0.0105	0.2799	1.0736
		*θ*	0.0988	1.0429	4.0505	95.80%	0.0212	0.3626	1.3178
	80	*α*	0.0743	0.4182	1.6002	95.20%	-0.0083	0.1658	0.6267
		*β*	0.1077	1.0010	3.8360	96.40%	-0.0017	0.2642	1.0425
		*δ*	0.1918	0.6324	2.2098	97.20%	0.0155	0.1806	0.6680
		*θ*	-0.0113	0.7146	2.8034	94.40%	-0.0068	0.2239	0.8697
	150	*α*	0.0499	0.2923	1.1204	96.40%	-0.0041	0.0998	0.3927
		*β*	0.1029	0.7916	2.9992	95.60%	0.0007	0.1247	0.4835
		*δ*	0.1501	0.5318	1.8908	96.20%	0.0018	0.1019	0.3860
		*θ*	-0.0408	0.5904	2.3042	96.20%	-0.0054	0.1253	0.4770
3.5	30	*α*	0.1958	0.7921	2.9559	95.00%	-0.0008	0.2492	0.8705
		*β*	0.0601	1.9303	7.5291	98.20%	-0.0003	0.4393	1.7002
		*δ*	0.3755	1.1094	3.7505	98.20%	0.0204	0.3182	1.2019
		*θ*	0.1636	1.2586	4.8439	94.80%	0.0060	0.3538	1.3703
	80	*α*	0.1046	0.4331	1.6202	96.60%	0.0014	0.1506	0.5523
		*β*	0.0631	1.2014	4.6339	95.60%	-0.0044	0.2714	1.0539
		*δ*	0.2101	0.7126	2.5079	97.60%	-0.0026	0.1939	0.7319
		*θ*	0.0369	0.8250	3.2299	95.00%	0.0046	0.2208	0.8245
	150	*α*	0.0276	0.2396	0.9304	96.00%	-0.0017	0.0980	0.3961
		*β*	0.0088	0.8191	3.2118	95.20%	-0.0020	0.1274	0.5033
		*δ*	0.1439	0.5703	2.0711	96.20%	0.0049	0.1112	0.4405
		*θ*	0.0106	0.6275	2.4616	95.20%	-0.0065	0.1233	0.4875

**Table 3:** RB, MSE, and length of CI of OLINH distribution for MLE, and Bayesian when *β* = 1.5, *δ* = 1.5, *θ* = 1.5.

*β* = 1.5, *δ* = 1.5, *θ* = 1.5									
			MLE				Bayesaian		
*θ*	n		Bias	MSE	L.CI	CP	Bias	MSE	L.CI
0.5	30	*α*	0.0997	0.1983	0.7533	96.00%	0.0407	0.0956	0.3322
		*β*	0.2218	1.4176	5.4071	95.00%	0.0166	0.3915	1.5627
		*δ*	0.1974	0.6554	2.2944	96.80%	0.0210	0.3211	1.1880
		*θ*	0.0415	0.7196	2.8132	95.20%	0.0221	0.3806	1.4486
	80	*α*	0.0397	0.1033	0.3979	95.00%	0.0163	0.0568	0.2127
		*β*	0.0693	0.7226	2.8060	95.00%	-0.0081	0.2574	1.0337
		*δ*	0.1093	0.4118	1.4823	95.60%	0.0060	0.1929	0.7347
		*θ*	-0.0151	0.4188	1.6411	96.20%	0.0031	0.2292	0.8833
	150	*α*	0.0078	0.0666	0.2610	95.00%	0.0070	0.0385	0.1435
		*β*	0.0198	0.5127	2.0083	96.80%	0.0017	0.1280	0.4827
		*δ*	0.0854	0.3194	1.1482	95.40%	0.0049	0.1145	0.4576
		*θ*	-0.0413	0.3014	1.1576	94.00%	-0.0075	0.1235	0.4714
1.75	30	*α*	0.2290	1.1879	4.3881	94.40%	-0.0066	0.3415	1.3009
		*β*	0.2237	1.1951	4.5009	95.40%	-0.0027	0.3824	1.4830
		*δ*	0.2637	0.7904	2.6851	98.40%	0.0450	0.2828	1.0202
		*θ*	0.0208	0.8637	3.3867	95.40%	0.0020	0.3342	1.2597
	80	*α*	0.0851	0.6607	2.5258	94.60%	-0.0102	0.1937	0.7272
		*β*	0.1329	0.8555	3.2647	96.00%	-0.0055	0.2457	0.9412
		*δ*	0.1428	0.5255	1.8831	96.80%	0.0175	0.1675	0.6197
		*θ*	-0.0151	0.6154	2.4131	95.40%	-0.0074	0.2107	0.8207
	150	*α*	0.0911	0.5307	1.9864	94.20%	-0.0043	0.1200	0.4604
		*β*	0.1522	0.7020	2.6051	95.20%	0.0045	0.1377	0.5195
		*δ*	0.1005	0.4668	1.7334	97.80%	-0.0038	0.0987	0.3609
		*θ*	-0.0040	0.5595	2.1953	96.80%	0.0030	0.1230	0.4724
3.5	30	*α*	0.0880	1.5161	5.8248	95.80%	-0.0164	0.4089	1.5354
		*β*	0.0680	0.8528	3.3222	95.80%	0.0011	0.3622	1.3914
		*δ*	0.2331	0.7859	2.7617	97.80%	0.0393	0.2572	0.9488
		*θ*	0.0044	0.8943	3.5090	95.20%	0.0105	0.3298	1.2096
	80	*α*	0.0064	0.9096	3.5680	95.00%	-0.0069	0.2481	0.9660
		*β*	-0.0086	0.5052	1.9818	95.20%	-0.0041	0.2255	0.8644
		*δ*	0.1430	0.5293	1.8988	96.20%	0.0164	0.1571	0.5881
		*θ*	-0.0257	0.5943	2.3272	95.60%	0.0002	0.2071	0.7571
	150	*α*	0.0369	0.9408	3.6565	95.00%	-0.0012	0.1302	0.4980
		*β*	0.0456	0.5366	2.0886	96.20%	-0.0060	0.1297	0.4906
		*δ*	0.1113	0.4687	1.7186	97.20%	0.0054	0.0922	0.3519
		*θ*	0.0092	0.6261	2.4562	97.20%	0.0021	0.1158	0.4301

## 6 Analysis of COVID-19 vaccination

COVID-19 vaccination rate data from 46 different countries in southern Africa is considered, some statistical measures are summarized in Table 4. Our goal is to model these rates by implementing the OLINH distribution to describe their trend and to predict future values of the vaccination rate. For that purpose some goodness of fit measures are used and a comparison between our model and other competitive models are presented in Table 5. The goodness of fit measures are: Kolmogorov-Smirnov statistics (KSS) with P-value (KSP-value), Cramér-von Mises statistics (CVMS), Anderson-Darling statistics (ADS), Akaike information criterion statistics (AICS), Bayesian information criterion statistics (BICS), Hannan-Quinn information criterion statistics (HQICS) and consistent AICS (CAICS).

**Table 4:** Minimum, first and third quarterly, median, mean and variance for COVID-19 vaccination.

Min.	1st Qu.	Median	Mean	3rd Qu.	Max.	var
0.0420	1.1190	3.1710	9.8037	12.5448	72.2860	256.9492

**Table 5:** MLE estimates, SE, and different measures for COVID-19 vaccination.

		estimates	SE	CVMS	ADS	KSS	KSP-value	AICS	CAICS	BICS	HQICS
OLINH	*α*	9.0385	4.1244	0.0501	0.3185	0.0856	0.8606	292.1784	293.1540	299.4930	294.9185
	*β*	1.8521	0.9203								
	*δ*	716.7624	66.2961								
	*θ*	0.2115	0.1034								
EL	*α*	1.0330	0.4006	0.0638	0.3782	0.1070	0.6294	292.7365	293.3080	299.5222	295.7916
	*β*	1.3510	0.6870								
	*δ*	5.1071	6.1123								
KW	*α*	1.3645	0.9455	0.0993	0.6261	0.1276	0.4084	297.3417	298.3173	304.6563	300.0818
	*β*	0.7670	0.0020								
	*δ*	1.4914	0.4256								
	*θ*	0.1863	0.0257								
KITL	*α*	0.6727	0.0338	0.0554	0.3824	0.1058	0.6433	293.3680	293.9395	299.8540	295.4231
	*β*	0.0905	0.0133								
	*δ*	9.0592	0.0122								
INH	*δ*	0.3538	0.0561	0.0545	0.3393	0.0890	0.8283	293.0967	293.3757	300.7540	295.4667
	*θ*	11.1900	5.0294								
OWITL	*α*	0.7249	0.1680	0.0545	0.3689	0.0875	0.8423	292.9472	293.5186	299.6433	298.0023
	*β*	0.7639	0.5142								
	*δ*	0.6648	0.3838								
NEF	*α*	101.8728	13.3171	0.0546	0.3366	0.0916	0.8018	292.4949	293.8066	299.9809	295.5500
	*β*	8.2935	3.4193								
	*δ*	0.2780	0.1416								
WL	*α*	29.2803	2.1566	0.0539	0.3292	0.0994	0.7165	292.4813	293.4569	299.7959	295.2214
	*β*	2.9211	0.5771								
	*δ*	0.0538	0.0146								
	*θ*	0.0361	0.0390								
EOWINH	*α*	2.8130	0.5873	0.0549	0.3347	0.0985	0.7264	293.8822	294.8578	301.1968	296.6223
	*β*	2.1734	2.0397								
	*δ*	0.1565	0.1711								
	*θ*	64.0368	14.5102								
MKINH	*α*	5.6926	3.4110	0.0547	0.3299	0.0910	0.8073	292.2558	293.8272	299.7417	295.3108
	*δ*	771.0487	64.8183								
	*θ*	0.2142	0.1024								

The considered data belong to 46 Countries in southern Africa, as following: Saint Helena, Nigeria, Seychelles, Democratic Republic of the Congo, Mali, Malawi, Madagascar, Mauritius, South Sudan, Equatorial Guinea, Burkina Faso, Mauritania, Botswana, Cabo Verde, Ethiopia, Guinea-Bissau, Ivoire, Liberia, Algeria, Mozambique, Chad, Gambia, Kenya, Comoros, Guinea, Central African Republic, Congo, Eswatini, Namibia, Benin, Niger, Uganda, United Republic of Tanzania, South Africa, Senegal, Angola, Cameroon, Zambia, Ghana, Rwanda, Zimbabwe, Sierra Leone, Lesotho, Togo, Sao Tome and Principe, and Gabon.

The data represents the rate of persons fully vaccinated per 100 as follows: 0.042, 0.205, 0.285, 0.319, 0.464, 0.550, 0.889, 0.895, 0.939, 0.986, 1.000, 1.088, 1.212, 1.244, 1.450, 1.593, 1.844, 2.039, 2.157, 2.167, 2.334, 2.440, 2.657, 3.685, 3.879, 4.493, 4.800, 4.944, 5.155, 5.674, 7.602, 10.004, 12.238, 12.520, 12.553, 13.063, 15.105, 15.229, 15.629, 15.848, 18.641, 18.940, 29.885, 58.162, 61.838, and 72.286.

**Fig 5:**
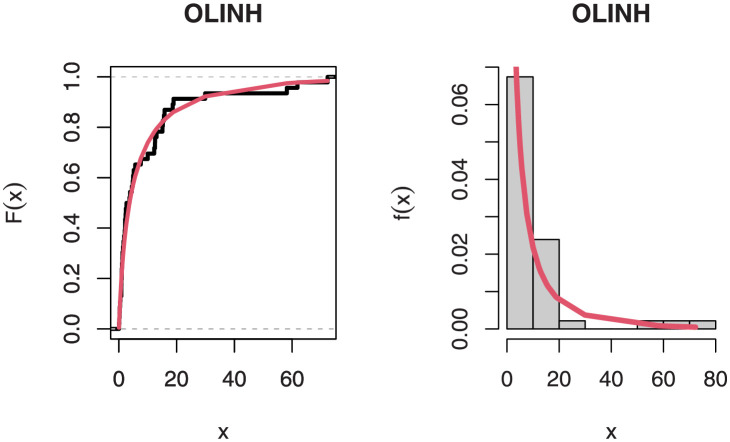
Estimated CDF and *pdf* of the OLINH distribution for COVID-19 vaccination data.

**Fig 6:**
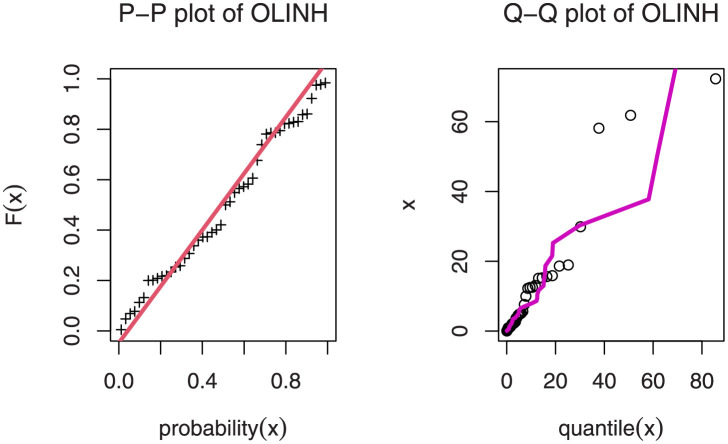
P-P and Q-Q plot of the OLINH distribution for COVID-19 vaccination data.

**Fig 7:**
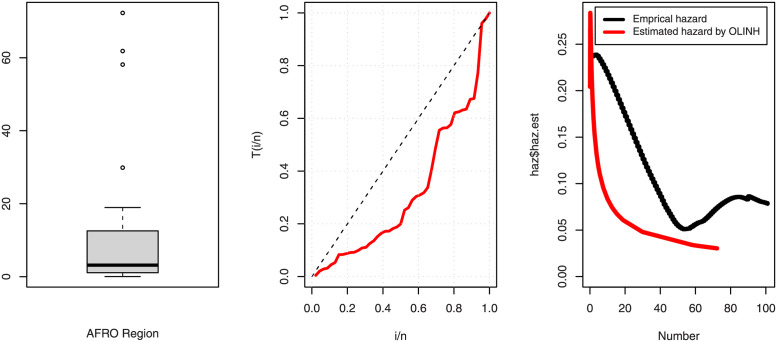
Boxplot, TTT plot and estimated hazard of COVID-19 vaccination data.

**Fig 8:**
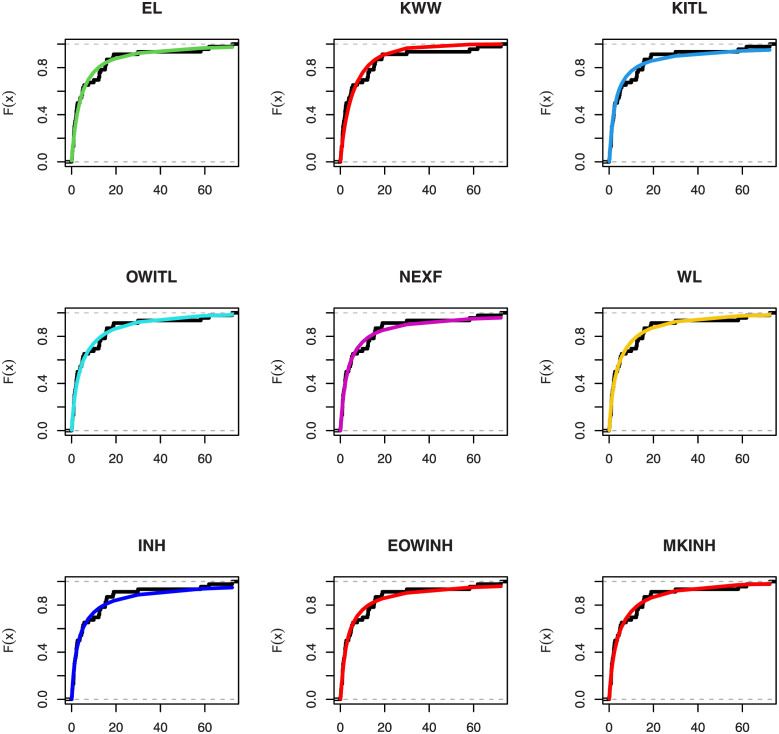
Estimated CDF for different models of COVID-19 vaccination data.

**Fig 9:**
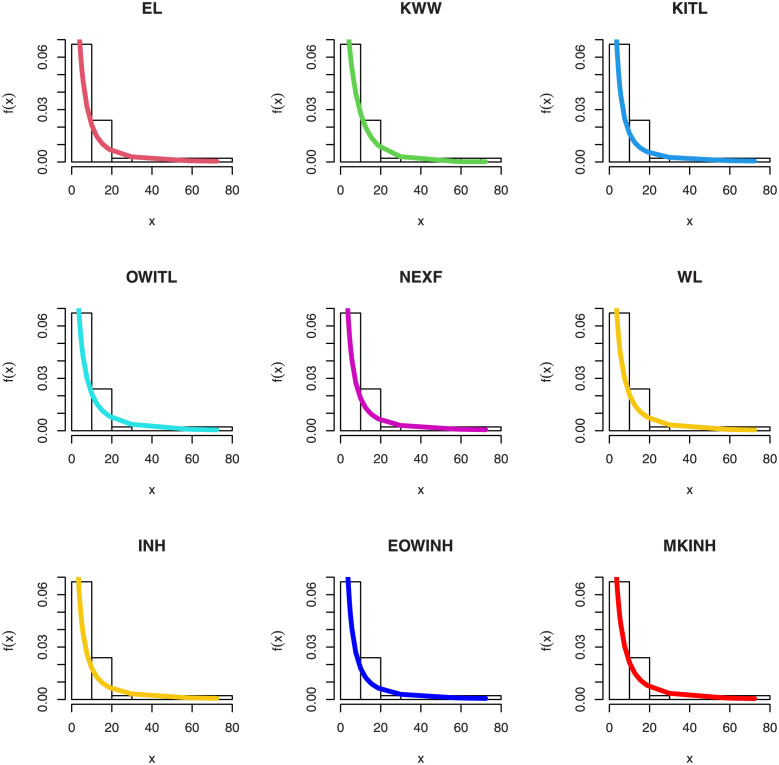
Estimated *pdf* for different models of COVID-19 vaccination.

**Fig 10:**
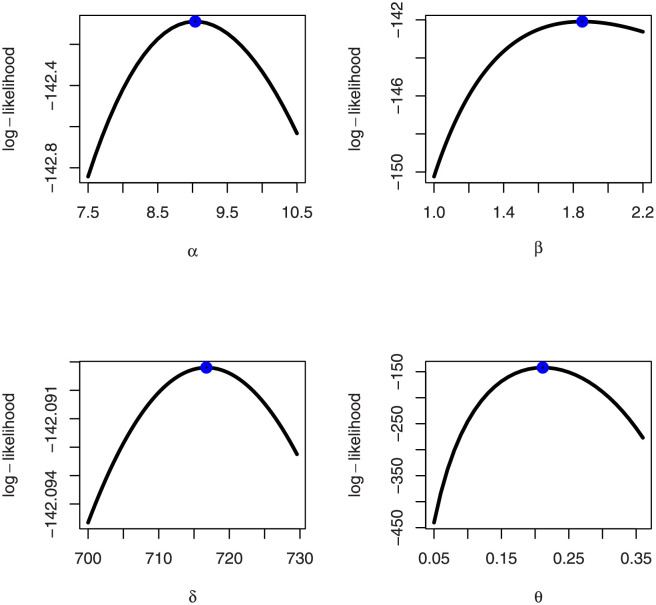
Log-likelihood value with parameters values of the OLINH distribution for COVID-19 vaccination data.

**Fig 11:**
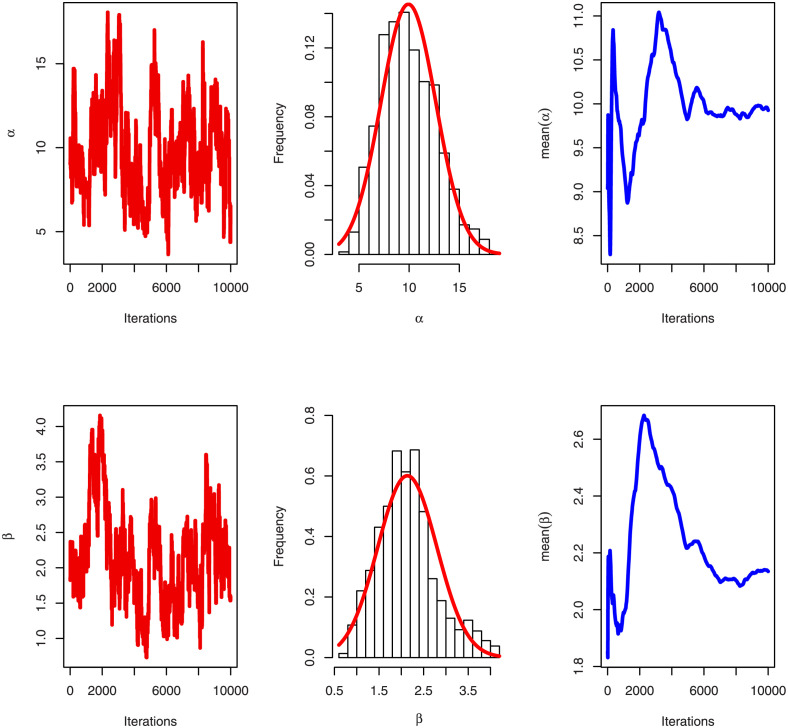
Convergence of MCMC estimation of OLINH distribution for COVID-19 vaccination for the parameters *α*, and *β*.

**Fig 12:**
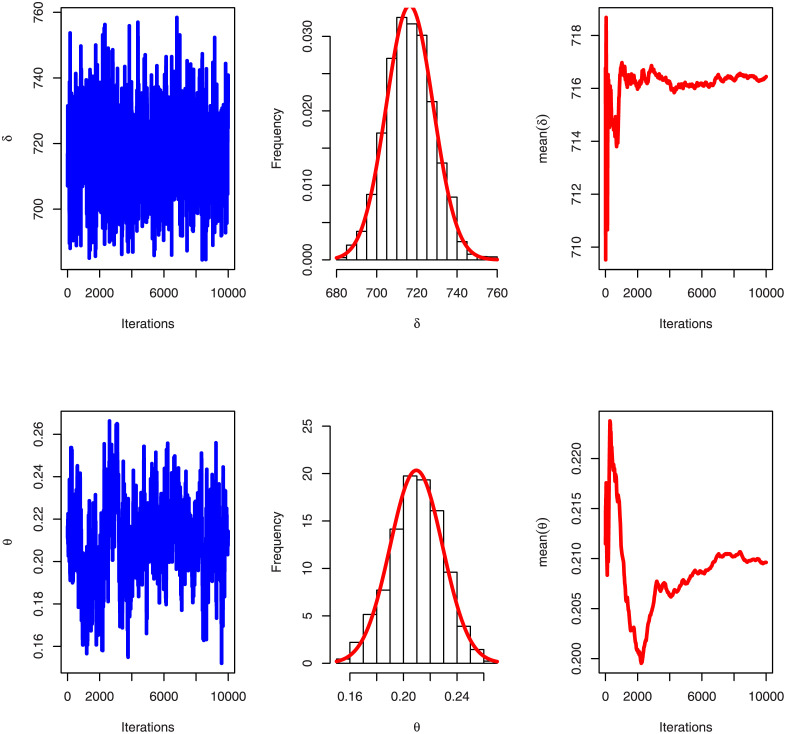
Convergence of MCMC estimation of OLINH distribution for COVID-19 vaccination for the parameters δ, and θ.

**Fig 13:**
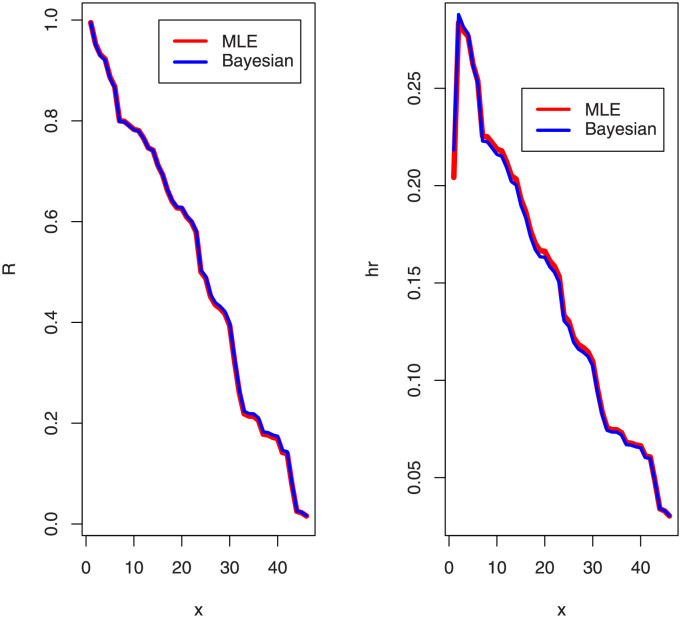
Estimated survival function and the hazard rate of OLINH distribution for COVID-19 vaccination data.

Table 5 shows that the OLINH distribution has the least values for all information measures with respect to other distributions. The suggested competitive distributions are: the extended odd Weibull inverse Nadarajah-Haghighi (EOWINH)(Almetwally [24]), exponential Lomax (EL) (El-Bassiouny et al. [25]), Kumaraswamy Weibull (KW) (Cordeiro et al. [26]), Kumaraswamy Inverted Topp-Leone (KITL) (Hassan et al. [10]), odd Weibull inverse Topp-Leone (OWITL) (Almetwally [27]), new exponential-X Fréchet (NEF) (Alzeley et al. [28]), Modified Kies INH (MKINH), and Weibull Lomax (WL) (Tahir et al. [29]). As a result, we conclude that OLINH best suits and fit the COVID-19 vaccination rate data set. Fig 5 shows the OLINH estimated CDF and *pdf* of the COVID-19 vaccination data. Fig 6 shows the PP-plot, and QQ-plot of fitted OLINH of the COVID-19 vaccination data. The Q-Q and P-P plots in Fig 6 indicate that our distribution is a good fit for modeling the actual data. Fig 7 shows Box plot, TTT plot and estimated hazard with empirical hazard. Fig 8 represents the estimated CDF with empirical CDF for different models of COVID-19 vaccination. Fig 9 shows estimated *pdf* with probability in histogram for different models of COVID-19 vaccination. The Bayesian estimation method of the OLINH distribution is the best estimation method, according to Table 6. Fig 10 show the estimates values have maximum of log-likelihood values of OLINH distribution. Figs 11 and 12 depicts history plots, estimated marginal posterior density, and MCMC convergence of *α*, *β*, *δ* and *θ*. Fig 13 show estimated survival and and hazard rate by the MLE and the Bayesian estimation methods.

**Table 6:** MLE, and Bayesian estimates, SE of OLINH distribution for COVID-19 vaccination.

	MLE				Bayesian			
	estimates	SE	Lower	Upper	estimates	SE	Lower	Upper
*α*	9.0385	4.1244	0.9547	22.7255	9.9268	2.7435	5.1000	15.1619
*β*	1.8521	0.9203	0.0483	4.6616	2.1348	0.6641	0.9533	3.6020
*δ*	716.7624	66.2961	586.8220	1573.1849	716.4462	11.6813	694.5620	738.8099
*θ*	0.2115	0.1034	0.0089	0.5166	0.2096	0.0196	0.1690	0.2464

## 7 Conclusion

A new Extension of INH and Lomax distributions called OLINH distribution is formulated in this paper. We studied its statistical properties and obtained its *pdf* as linear representation, quantile function of moments, moment generation functions, and Rényi entropy are also obtained. Point estimation of the OLINH unknown parameters *α*, *β*, *δ*, and *θ* were considered by the MLE, and the Bayesian estimation methods. Interval estimation of the OLINH parameters *α*, *β*, *δ*, and *θ* were considered by the MLE asymptotic approximation, and Bayesian credible interval estimation methods. To distinguish the performance of the different estimation methods, a comparison was carried out through Monte-Carlo simulation analysis using the R package. For that reason, the COVID-19 data sets were also considered, and OLINH was shown to match these data better compared to other competitive distributions. Bayesian estimation was better than the MLE for estimating the parameters of OLINH distribution.
